# The Role of Purinergic Signaling in Heart Transplantation

**DOI:** 10.3389/fimmu.2022.826943

**Published:** 2022-04-21

**Authors:** Yanzhi Jiang, Jianxin Lin, Haiyun Zheng, Ping Zhu

**Affiliations:** Guangdong Cardiovascular Institute, Guangdong Provincial People’s Hospital, Guangdong Academy of Medical Sciences, Guangzhou, China

**Keywords:** purinergic signaling, ATP, adenosine, ischemia reperfusion injury, heart transplantation

## Abstract

Heart transplantation remains the optimal treatment option for patients with end-stage heart disease. Growing evidence demonstrates that purinergic signals mediated by purine nucleotides and nucleosides play vital roles in heart transplantation, especially in the era of ischemia-reperfusion injury (IRI) and allograft rejection. Purinergic signaling consists of extracellular nucleotides and nucleosides, ecto-enzymes, and cell surface receptors; it participates in the regulation of many physiological and pathological processes. During transplantation, excess adenosine triphosphate (ATP) levels are released from damaged cells, and driver detrimental inflammatory responses largely *via* purinergic P2 receptors. Ecto-nucleosidases sequentially dephosphorylate extracellular ATP to ADP, AMP, and finally adenosine. Adenosine exerts a cardioprotective effect by its anti-inflammatory, antiplatelet, and vasodilation properties. This review focused on the role of purinergic signaling in IRI and rejection after heart transplantation, as well as the clinical applications and prospects of purinergic signaling.

## Introduction

Heart transplantation is the ideal therapeutic approach for patients with end-stage heart disease (1), with the number of heart transplants performed each year continuing to increase globally throughout the past decade (2). Due to advances in surgical techniques, organ preservation methods, and application of novel immunosuppressants, tremendous progress has been achieved in the field of heart transplantation. The International Society of Heart and Lung Transplantation reported that, currently, more than 6,000 heart transplants are performed worldwide each year (3). Additionally, according to the latest data from 481 adult heart transplant centers and 210 pediatric heart transplant centers around the world, the median survival times of adult and child heart recipients are 12.1 years and 24.5 years, respectively (3–5). However, severe complications, such as rejection, infection, and post-operative malignancy have severely hindered the development of heart transplantation as a treatment option, with an annual mortality rate of approximately 3–4% (6). Furthermore, the heart is more vulnerable to ischemia-reperfusion injury (IRI) than the liver or kidney, which limits clinical preservation time to 4–6 hours, resulting in a critical shortage of donor hearts (7). Therefore, a better understanding of the factors that negatively affect heart transplantation is of utmost importance.

**Figure 1:**
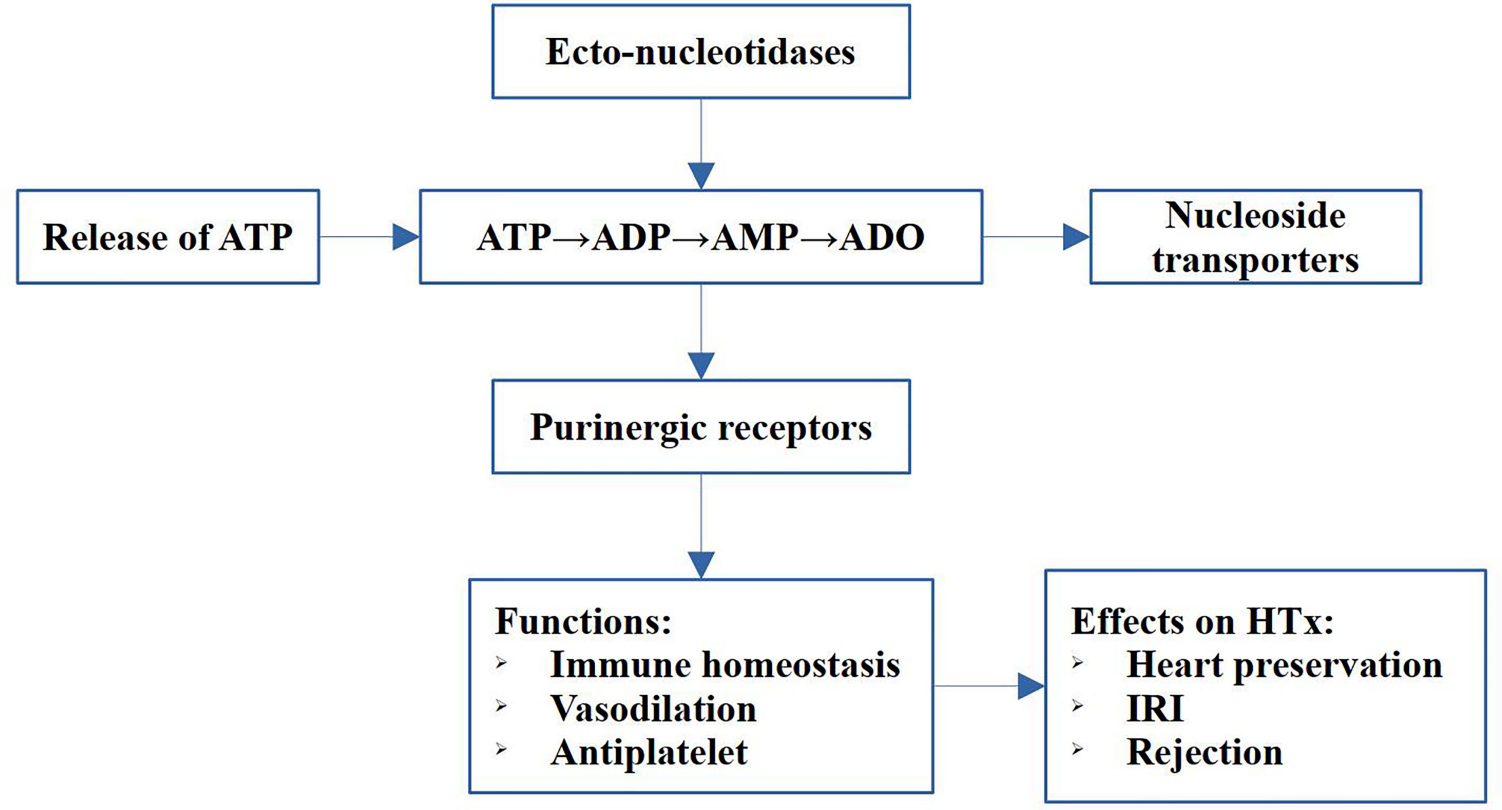
Schematic diagram of the strategy of the study. The main components of the purinergic signaling includes release of ATP, the hydrolysis of ATP to adenosine, the ecto-nucleotidases, transporters and purinergic receptors. The purinergic signaling exert immune regulation, vasodilation and antiplatelet functions, and play pivotal roles in heart preservation, IRI and rejection in heart transplantation (HTx).

Purinergic signaling is a kind of evolutionarily conserved communication pathway between cells, and participates in the regulation of many physiological and pathological processes (8, 9). It consists of extracellular nucleotides and nucleosides, ecto-enzymes, and cell surface receptors (10). Recent evidence shows involvement of these nucleotides and cell surface receptors in both IRI and rejection in heart transplantation (11). In addition, studies have indicated that purinergic signaling elements were potential targets for preventing inflammation and rejection. Specifically, adenosine plays a significant role in the diagnosis and treatment of complications following heart transplantation. The strategy of the study was illustrated in **Figure 1**. In this review, we introduced the purinergic signaling molecules, the ecto-nucleotidases, the purinergic receptors and their role in IRI and rejection after heart transplantation. Briefly, adenosine triphosphate (ATP) promotes acute injury and inflammation after heart transplantation, whereas adenosine has anti-inflammatory and protective effects in IRI and donor heart preservation, as well as function on immune cells and promote tolerance after transplantation. Therefore, balancing ATP and adenosine using ATP hydrolysis, modulating purinergic receptors, and increasing adenosine level are promising strategies for reducing posttransplant inflammation, rejection, and graft failure and prolonging the graft survival. This review aimed to present in-depth information on purinergic signaling in heart transplantation.

## Purinergic Signaling Molecules

**Figure 2:**
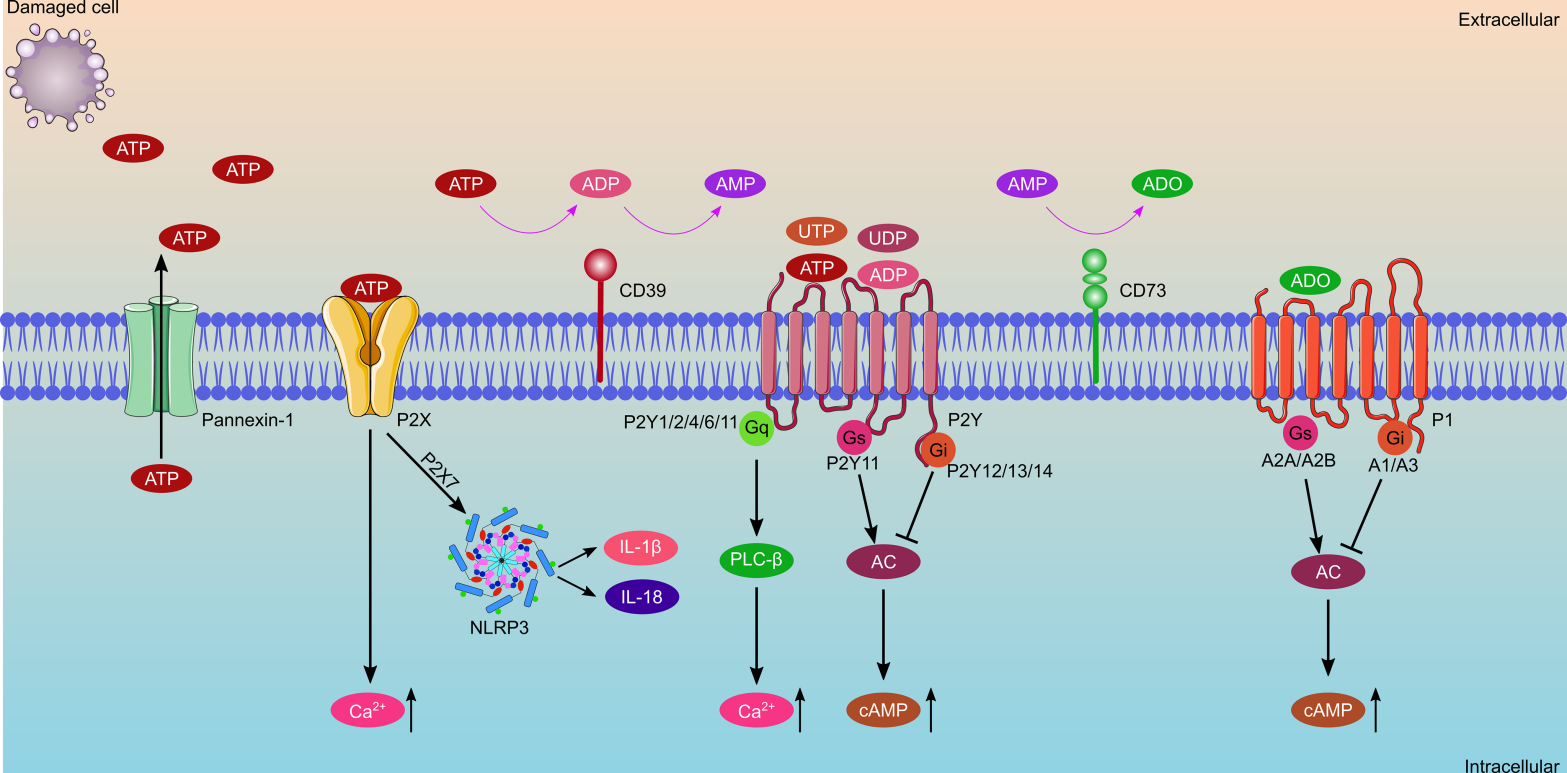
Schematic diagram of purinergic signaling components, which consist of extracellular nucleotides and nucleosides, ecto-enzymes, and cell surface receptors. ATP is released from damaged cells or through the Pannexin-1 channel, and then is rapidly dephosphorylated to ADP and AMP by CD39, with AMP further catalyzed to adenosine by CD73. Purinergic receptors consist of P1 receptors and P2 receptors. P1 receptors bind with adenosine, which consist of A1, A2A, A2B and A3 receptors. A1Rs and A3Rs are coupled with Gi proteins and inhibit adenylate cyclase, whereas A2ARs and A2BRs coupled with Gs proteins and stimulate adenylate cyclase. P2 receptors consist of P2X and P2Y receptors, of which, P2XRs bind with ATP only, while P2YRs can bind with ATP, ADP, UTP, and UDP. Activation of P2XRs increases the concentration of intracellular Ca^2+^. Activation of P2YRs causes a change in the concentration of intracellular Ca^2+^ or cAMP.

Extracellular purinergic signaling molecules, consisting of ATP, adenosine diphosphate (ADP), and adenosine (**Figure 2**), play significant roles in the transplantation process. In this process, ATP is an important priming factor. ATP was first conceived as a neurotransmitter in 1972 (12); it gradually gained further acceptance as different receptor subtypes were discovered (13). Currently, ATP has been shown consistently to be an important extracellular ligand for autocrine signal transduction, cell-to-cell communication, and neurotransmission (10). ATP is released during cell lysis or by means of specialized ATP release mechanisms involving exocytosis, transporters (such as ATP-binding cassette transporter superfamily) or ATP-permeable channels. Extracellular ATP (eATP) levels are minimal under normal physiological conditions. However, large amounts of ATP and ADP are released when cells or tissues are damaged during IRI or during acute rejection after transplantation. Additionally, inflammation-induced ATP released by endothelial cells and platelets overwhelms ATP metabolism. High levels of ATP are important damage-associated molecular patterns (DAMPs) that promote chemotaxis and excitation of immune cells within hours (14). Ecto-nucleosidases rapidly dephosphorylate eATPs to ADP, adenosine monophosphate (AMP), and finally adenosine (15–17). Adenosine molecules are transported intracellularly or extracellularly, once they are synthesized by nucleoside transporters, including the concentrative nucleoside transporters (CNTs) and the equilibrative nucleoside transporters (ENTs). CNTs comprise three members (CNT1-3) and facilitate adenosine transport against its concentration gradient, both intracellularly and extracellularly (18). ENTs, including ENT1-4, which transport adenosine across the cell membrane, depend on the concentration gradient (19). Adenosine molecules function by cytoprotection and protect cell damage from ischemia and hypoxia. Moreover, adenosine is widely used as antiarrhythmic drugs and an important component of organ preservation solutions for heart transplantation.

## Ecto-Nucleotidases

Ecto-nucleotidases are indispensable key factors in the process of purinergic signal transduction. They regulate the hydrolysis of ATP to ADP, AMP, and adenosine, thereby controlling the balance of ATP and adenosine (**Figure 2**). The primary ecto-nucleotidases involved in regulating the process of ATP hydrolysis to adenosine include the ecto-nucleoside triphosphate diphosphohydrolase family (E-NTPDases), ecto-5’-nucleotidase (NT5E/CD73), ecto-nucleotide pyrophosphatases (E-NPPases), adenosine deaminase, NAD glycohydrolase, CD38/NADase, alkaline phosphatase (AP), adenylate kinase (AK), and nucleoside diphosphate kinase (10). E-NTPDases are widely distributed in all tissues and contain eight members (E-NTPDase1-8). Four E-NTPDases (1–3, 8) are surface-located; among which, CD39 (NTPDase 1) is the most representative ecto-nucleotidases for ATP hydrolysis. CD39 is primarily expressed on lymphocytes and resting vascular endothelial cells, and its main function is to break down extracellular ATP into ADP and AMP. CD73, mainly expressed on follicular dendritic cells (DCs), T lymphocytes, and B lymphocytes, further catalyzes extracellular AMP into adenosine. Finally, adenosine can be degraded into inosine by adenosine deaminase or transported into the cell by a nucleoside transporter (20–22). Previous studies have revealed that the combination of CD39 and CD73 converts ATP to adenosine much faster than E-NTPDase2/CD73 and E-NTPDase8/CD73. Therefore, the theory of the CD39-adenosinergic axis was proposed (23, 24). In the field of heart transplantation, CD39 and CD73 are vital regulators of eATP and adenosine balance, and the shift in the conversion of ATP to adenosine is important for anti-inflammation, suppression of organ transplantation rejection, and promotion of graft survival (25). Zhong et al. (26) engineered CD39/CD73 bifunctional fusion proteins, which are expected to be a therapeutic agent for scavenging ATP and producing adenosine for anti-inflammatory and immunomodulatory functions. E-NPPs have several substrates, including ATP, ADP, NAD+, and lysophosphatidylcholine (LPC). Currently seven members (NPP1-7) have been identified, among which NPP1 and NPP2 were well investigated. NPP1 is mainly expressed on inflammatory cells, whereas NPP2 is expressed on inflammatory and tumor cells. The main function of NPP2 is to convert LPC to lysophosphatidic acid (LPA) (27). APs are the only ectoenzymes that can hydrolyze ATP, ADP, and AMP. Currently, four APs have been identified, and they are named according to their tissue distribution characteristics: placental AP (PLAP), germ-cell AP, intestinal AP (IAP), and the tissue-nonspecific form of AP (TNAP). TNAP is mainly involved in bone mineralization, and disturbance of phosphate metabolism in chronic kidney disease (28, 29). In addition, AK is the critical enzyme responsible for cellular adenine nucleotide homeostasis through the catalysis of the reaction 2ADP ↔ ATP + AMP. It is also involved in extracellular adenine nucleotide metabolism by minimal ecto-expression or released from cells into the extracellular space (30).

## The Purinergic Receptors

Purinergic receptors are distributed widely throughout different organs, such as the brain, kidney, heart, and blood vessels. Part of earlier work examining extracellular purinergic signaling was performed on the cardiac system. Purinergic receptors in the heart are primarily distributed in the myocardium and coronary vascular smooth muscle, as well as cardiac adrenergic and cholinergic nerve terminals (31). ATP binds to purinergic receptors once they are released into the extracellular space. Purinergic receptors currently consist of two main families: adenosine receptors (P1 receptors) and purine nucleotide (ATP and ADP) receptors (P2 receptors) (**Figure 2**) (10). P1 receptors were first characterized and cloned in the early 1990s (32). They are a class of G-protein-coupled receptors, including A1, A2A, A2B, and A3 receptors (33). P2 receptors consist of two subtypes: the ion-channel receptor P2X and G-protein-coupled receptors P2Y (P2Y1, P2Y2, P2Y4, P2Y6, P2Y11, P2Y12, P2Y13, and P2Y14) (34, 35). Generally, eATP induces an inflammatory response through P2 receptors, such as P2X7 and P2Y2 receptors. Conversely, levels of adenosine rise along with ATP hydrolysis, which exerts anti-inflammatory functions through P1 receptors such as A2A and A2B receptors (36–38).

P1 receptors contain four receptor subtypes that have been named by the order of their discovery as A1, A2A, A2B, and A3 receptors. Among these receptors, A1 and A2A receptors are far more sensitive to adenosine and its agonists than the others, as the former works in the nanomolar range, whereas the latter works in the micromolar range (39). P1 receptors are G-protein-coupled receptors that are composed of two domains—the extracellular domains (N-terminus) that comprise specific glycosylation sites and extracellular loops, and the intracellular domains (C-terminus) with phosphorylation and palmitoylation sites and intracellular loops (33). A1 and A3 receptors are coupled with Gi proteins, and inhibit adenylate cyclase. Conversely, A2A and A2B receptors are coupled with Gs proteins, and stimulate adenylate cyclase (40). Recent studies have demonstrated that A1 receptors are involved in renal fibrosis (41), hepatocyte glucose metabolism (42), cerebral ischemia-reperfusion-induced cognitive impairment (43), and chronic heart failure (44). A2A receptor agonists had a protective effect on IRI in the kidney (45), brain (46), liver (47) and heart (48). Additionally, A2A receptor agonists were used to inhibit COVID-19-induced lung inflammation and thrombogenesis (49). Both A2B and A3 receptors have been reported to have protective effects on IRI (50–52) and show infarct-sparing effects in a myocardial infarction model in mice (53). Additionally, A2B receptors have been identified as biomarkers for lung cancer diagnosis and prognosis (54), whereas A3 receptors have been shown to participate in tumorigenesis and chemotherapy (55, 56).

P2X receptors are ligand-gated receptors and consist of seven subtypes (P2X1–7) (57). Generally, P2X receptors show a lower affinity for ATP than P2Y receptors (36). A higher concentration of ATP is needed to open channels (58). ATP binding with P2X receptors causes Na^+^, K^+^, and Ca^2+^ cations to flow across the cell membrane, resulting in specialized functions (59). Low levels of P2X1 receptors were detected on cardiac myocytes, with P2X1 receptors mainly expressed on vascular smooth muscle, causing arterial contractions when activated by ATP (60, 61). Additionally, P2X1 receptors are expressed on platelets, mediating shape changes, and resulting in aggregation (62). Furthermore, P2X1 receptors have been reported to regulate IL-22 and are involved in efficient liver regeneration (63). P2X3 and P2X4 receptors are involved in myocardial ischemic injury and neuropathy in type 2 diabetes (64–67). Whereas few studies have examined P2X5 and P2X6 receptors, several studies have explored how P2X7 receptors promote IRI and play an important role in immunity and inflammation (68).

P2Y receptors are metabotropic receptors that can bind with ATP, ADP, uridine triphosphate (UTP), and uridine diphosphate (UDP). Some P2Y receptors (P2Y1, P2Y2, P2Y4, P2Y6, and P2Y11) couple with Gq proteins and activate phospholipase C-β, leading to an increase in intracellular Ca^2+^ level; whereas others (P2Y12, P2Y13, and P2Y14) couple with Gi proteins and inhibit adenylyl cyclase, resulting in a decrease in cAMP level (69). P2Y receptors play a wide range of regulatory roles, such as in tumor progression (70), platelet aggregation and thrombosis (71), immune response (72), obesity and metabolism (73), pain transmission (74), organ fibrosis (75), and IRI (76). P2Y receptors have been shown to be activated by DAMPs, such as ATP and UTP, and recruit surrounding neutrophil granulocytes to mediate the inflammatory response. Conversely, P2Y receptors on phagocytes upregulate and help to clear apoptotic cells (77–79). P2Y receptors are involved in bacterial, viral, and parasitic infections. Although P2Y12 receptors were reported protective in sepsis; however, other studies have indicated that P2Y12 receptors induce lung injury (80–82). Furthermore, P2Y2 receptors have been reported to be cardioprotective, because activation of P2Y2 receptors causes reduced inflammation, which, in turn, reduces infarct size and improves cardiac function (83).

Currently, P2Y receptors that are reportedly involved in myocardial infarction include P2Y1, P2Y4, P2Y6, P2Y11, and P2Y13 receptors (82, 84–88). P2Y receptors have been thoroughly studied in thrombosis, among which, P2Y1 and P2Y12 receptors expressed on platelets can be activated by eATP and ADP, leading to the recruitment of platelets to the thrombus. The P2Y12 receptors are key players in thrombosis, and inhibition of P2Y12 receptors is widely used as a clinical thrombosis prevention strategy, with primary preparations, such as clopidogrel and ticagrelor (89, 90).

## Effect of Purinergic Signaling on IRI

**Figure 3:**
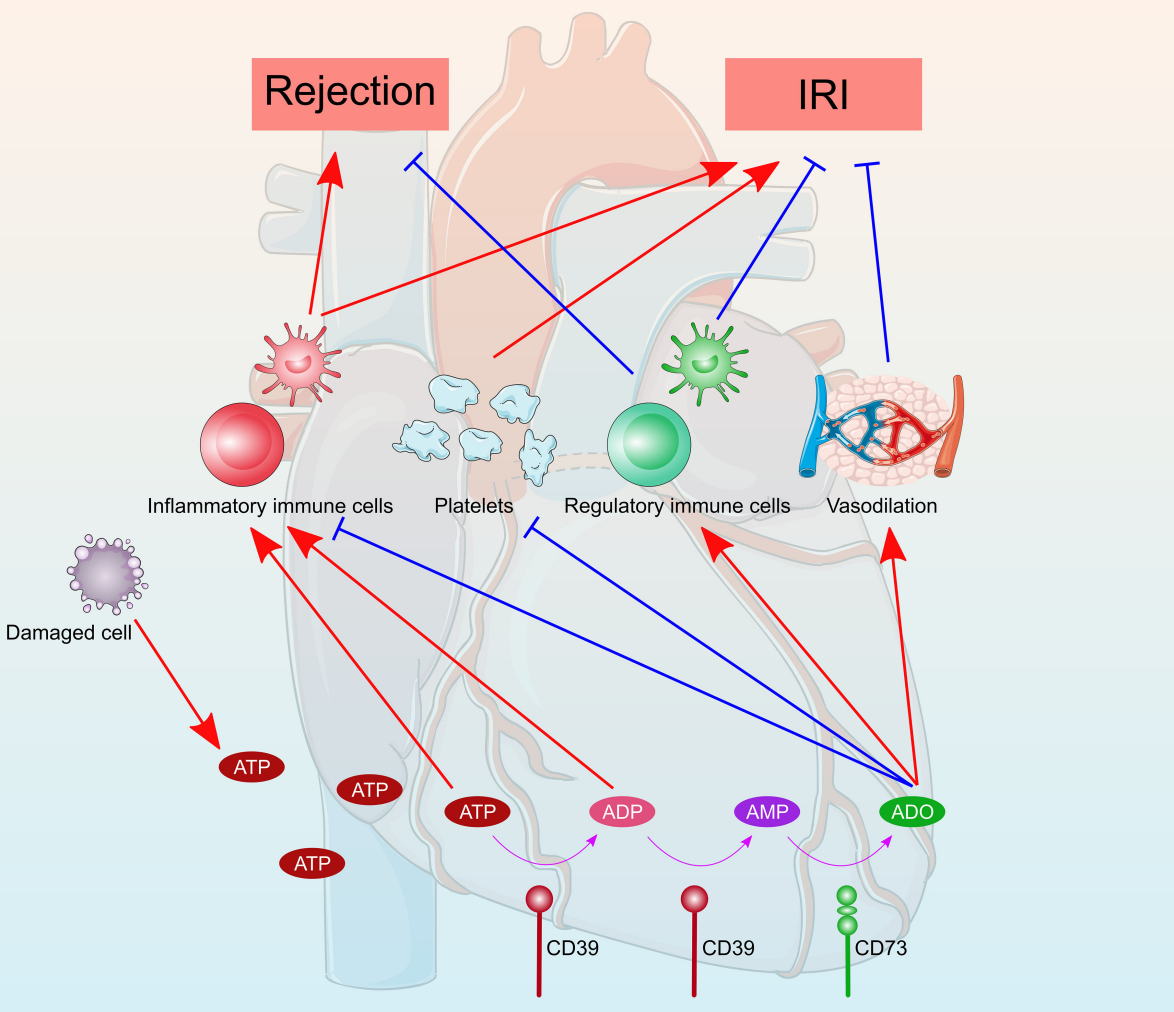
Schematic diagram of the role of purinergic signaling in heart transplantation. ATP were released from damaged cells during the transplantation process. ATP were converted into ADP and AMP by CD39 and then catalyzed to adenosine by CD73. Extracellular ATP and ADP promote inflammatory responses and exacerbate detrimental rejection and cardiac ischemia reperfusion injury. Whereas, adenosine generally has anti-inflammatory and immunosuppressive properties, which protect the heart from rejection and ischemia reperfusion injury. Furthermore, the antiplatelet and vasodilatory effects of adenosine attenuate heart rejection and ischemia reperfusion injury.

IRI is an inevitable event that occurs during heart transplantation, leading to delayed graft function, rejection, and decreased graft survival. Donor hearts are particularly more vulnerable to IRI than livers and kidneys, which can only be safely preserved for 4–6 hours using principally static cold storage methods in clinical practice (91). Purinergic signaling plays a vital role in IRI (**Figure 3**). During ischemia, cellular ATP is progressively exhausted and resynthesized *via* aerobic metabolism, which produces a lower level of ATP and rapidly leads to acidosis and necrosis of the myocardium (92). The resultant low level of cellular ATP cannot maintain cellular ion and membrane homeostasis, leading to cell death. During reperfusion, cellular ATP is recovered, accompanied with a reactive oxygen species burst and Ca^2+^ overload, causing cell damage and subsequent release of DAMPs and inflammatory responses. Extracellular ATP is a ubiquitous and an extremely efficient DAMP molecule that promotes inflammation following IRI (93, 94). Extracellular ATP exacerbates detrimental inflammatory responses largely through purinergic P2 receptors on the surface of immune cells (95). P2X7 receptors are mostly involved in inflammatory processes among purinergic P2 receptors, which triggers NLRP3 inflammasome activation and subsequent release of proinflammatory cytokines, such as IL-1β and IL-18 (96). P2X7 receptors mediate the NLRP3 inflammation pathway, promoting myocardial damage and cardiac fibrosis, thus leading to impaired cardiac function (97–100). Whereas ischemia preconditioning or postconditioning by activation of P2X7 receptors has been reported to be protective in cardiac IRI, where the cardioprotective effect was facilitated through the release of sphingosine-I-phosphate and adenosine *via* pannexin-1 and the P2X7 receptor-formed channel (101–103). Contrary to P2X receptors, emerging evidence has revealed a cardioprotective role of P2Y receptors, which has been extensively reviewed elsewhere (76). Notably, P2Y12 receptor antagonists (i.e., antiplatelet agents) have shown promising myocardial protection independent of platelet antiaggregatory effects in cardiac IRI and translated clinical studies (104, 105).

The cardioprotective effect of adenosine has long been recognized, and all four adenosine receptors have been implicated. Adenosine is used as an additive in blood cardioplegia to induce a more rapid polarized cardiac arrest *via* the A1 receptors (106, 107). A polarized membrane potential during initial reperfusion may minimize intracellular Ca^2+^ overload and reduce cardiac IRI. Utilize of adenosine-lidocaine based cardioplegia or preservation solution showed superior preservatory effect of donation after circulatory death heart grafts in preclinical models (108–110). Additionally, adenosine is known to induce vasodilation by binding to A2A and A2B receptors (111–113). A2A and A2B receptor-induced vasodilation attenuates myocardial ischemia by increasing nutrients/oxygen supply and blood flow (114–116). In addition to vasodilation, A2B receptors contribute to cardioprotection by stabilizing the rhythm protein Per2 in an HIF‐dependent manner (114, 117). A1 receptors signaling protects ischemic hearts by limiting oxidant damage; however, the precise mechanism underlying A3 receptors-induced cardioprotection remains to be elucidated (118, 119). The ecto-nucleotidases CD39 and CD73, which convert ATP to adenosine, have been demonstrated to be protective in cardiac IRI (120–124). Furthermore, the antiplatelet effect of CD39 also plays a protective role in cardiac IRI by preventing thrombosis. A single-chain antibody-CD39 fusion protein, targeting activated platelets by specifically binding to activated glycoprotein (GP)IIb/IIIa, holds strong promise for effective protection from cardiac IRI (125). AK are abundant phosphotransferase enzymes that catalyze the interconversion of adenine nucleotides (ATP, ADP, and AMP), and thus regulate the adenine nucleotide homeostasis in different intracellular compartments. AK1 deficiency in the heart exacerbates cardiac IRI and compromises post-ischemic coronary reflow as a consequence of reduced adenosine (126, 127). Modest elevation of AK1 protects the heart against cardiac IRI by underpinning myocardial adenine nucleotides homeostasis.

## Effect of Purinergic Signaling on Heart Allograft Rejection

Allograft rejection, the process by which a recipient’s immune system recognizes and exerts immune response to the donor heart, is a major concern post-transplantation. Purinergic signaling plays a pivotal role in the alloimmune response (**Figure 3**), which is a complex process that results from the interplay among multiple different cell types, including lymphocytes, monocytes, macrophages, and dendritic cells. Following transplantation, ATP is rapidly released from damaged or stressed cells *via* Panx1 channels, vesicular release, or cell rupture. eATP acts as a “danger signal” to promote proliferation and activation of immune cells by binding to excitatory ATP receptors, including inotropic P2X receptor and metabotropic P2Y receptor subtypes. Several recent studies have recognized that eATP accumulation and subsequent purinergic signaling play significant roles in heart allograft rejection. Upregulation of P2X7 receptors on graft-infiltrating lymphocytes have been observed in cardiac‐transplanted humans and mice, and targeting of the P2X7 receptors with periodate-oxidized ATP promoted long-term cardiac transplant survival in murine cardiac transplantation models (128). Blocking P2X7 receptors signaling by oxidized ATP inhibited M2 macrophage infiltration, prevented transplant vasculopathy, and induced long-term heart allograft survival in a murine model of chronic rejection (129). Although eATP was considered to promote immune responses and allograft rejection, the protective effects of ATP receptors on allograft rejection have been reported. Loss-of-function mutation of P2X7 receptors disrupted NLR Family Pyrin Domain Containing 3 (NLRP3)-mediated Th2 programming, leading to excessive Th17 generation and subsequently poor cardiac allograft outcomes (68). Pharmacologic P2Y11 receptors stimulation protected heart allograft from ischemia/reperfusion and rejection injuries, and prolonged cardiac allograft survival (130, 131).

In the extracellular space, ecto-nucleotidase hydrolyzes ATP to ADP, and subsequently to AMP and adenosine. Adenosine binds to G protein–coupled P1 receptors, and generally have anti-inflammatory and immunosuppressive properties. Both CD39 and CD73 have been implicated in modulation of heart allograft rejection. Overexpression of CD39 or administration of soluble CD39 improved cardiac xenograft survival with reduced vascular thrombosis (132–134). In a mouse cardiac transplantation model, CD73 deficiency in either donors or recipients promoted inflammatory cascades, resulting in reduced cardiac allograft survival and vasculopathy development (135). Hu et al. (136) reported that CD73 expression was critical for mesenchymal-like endometrial regenerative cell-mediated cardiac allograft protection.

## Clinical Application and Prospects

Studies on the molecular mechanisms of extracellular nucleoside signals provide several therapeutic targets for human disease. Any participants of purinergic signaling, such as the main purines (ATP, ADP, AMP, and adenosine), key enzymes (CD39 and CD73), and purinergic receptors (four P1 receptors, seven P2X receptors, and eight P2Y receptors) could be potential targets for human disease treatment. One promising strategy is to target the release of ATP during damage. For example, blocking the Panx1 channel can reduce the release of ATP to limit the downstream inflammatory response and activation of immune cells. The Panx1 inhibitor carbenoxolone has been reported to improve islet transplantation outcomes (137), ameliorate acute pain of rats (138), and reduce brain and lung IRI (139, 140). However, studies on carbenoxolone in heart transplantation are currently unavailable. Another well investigated ATP release inhibitor is clodronate, which is reported to improve renal IRI (141) and induce skin allograft tolerance (142). Clodronate has been used for selective macrophage depletion, with results showing that clodronate was protective against heart transplant rejection (143). Another approach is to increase ATP degradation in addition to reducing ATP release. Hence, targeting CD39 and CD73 has a promising future, considering that CD39 and CD73 are two of the most important enzymes for ATP hydrolysis. Targeting CD39 exhibits anti-inflammatory and anti-thrombolytic effects, and treatment with soluble CD39 prolongs the survival of heart transplant by preventing thrombosis (20, 134, 144). Furthermore, the expression of CD73 increases anti-inflammatory cytokine levels, leading to endometrial regenerative cell-induced inhibition of cardiac allograft rejection (136). Moreover, purinergic receptors are potential therapeutic targets. For example, stimulating the P2Y11 receptors in mice has been shown to protect heart transplants from IRI and decrease immune rejection response (131). Targeting P2Y12 receptors is one of the most well-studied strategies for its critical role in antithrombosis. Several P2Y12 receptors antagonists, such as clopidogrel, ticlopidine, prasugrel, ticagrelor, and cangrelor, have been widely used in clinical applications (70, 145, 146).

Another promising strategy is to increase the level of circulating adenosine with exogenous adenosine or to selectively activate adenosine receptors, considering that adenosine plays a significant role in heart transplantation (**Table 1**). First, adenosine is an essential component of the University of Wisconsin solution (UW) and Institut Georges Lopez-1 (IGL-1) preservation solutions. Researchers have reported that the adenosine-containing Histidine-tryptophan-ketoglutarate (HTK) preservation solution has a better protective effect on myocardium than the standard HTK preservation solution, which prevents myocardial cell swelling and necrosis by reducing oxidative and nitrosative stress (147). Pre-treatment with adenosine prolongs donor heart storage and protects heart grafts from IRI (148, 149). Second, adenosine is a common antiarrhythmic drug, and low dose adenosine protects the transplanted heart from post-transplantation arrhythmias (150). Furthermore, a prospective clinical study was conducted to investigate the safety and efficacy of adenosine in supraventricular tachycardia after heart transplantation and the results showed that low doses of adenosine convert supraventricular tachycardia to a sinus rhythm of <140 beats/minute (NCT02462941) (151). Moreover, adenosine has been used in the diagnosis of coronary artery vasculopathy (CAV), a common complication after heart transplantation. Adenosine stress perfusion cardiac magnetic resonance imaging (MRI) is a safe and noninvasive method for the diagnosis of CAV after heart transplantation (152, 153). Adenosine or adenosine receptor agonist was used in several clinical trials as a vasodilator for stress echo in a stress MRI (NCT03231371, NCT03102125, NCT02597543, and NCT05081115). Other drugs targeting adenosine receptors have been investigated. For example, the partial adenosine A1 receptor agonist neladenoson bialanate has been commonly used in chronic heart failure treatment (154, 155). Adenosine A2A receptor agonist CGS21680 reduces the inflammatory response of lung transplantation (156, 157) and liver transplantation (47). It can as well significantly reduce the infarct area of isolated perfused mouse hearts (48).

**Table 1:** Clinical trials of purinergic signaling involved in heart transplantation.

ClinicalTrials.gov Identifier	Year	Drug	Conditions	Study Title
NCT02462941	2015	Adenosine	Sinus Bradycardia, Atrioventricular Block	Analysis of Adenosine on Sinus and Atrioventricular Nodal Conduction in the Pediatric Transplanted Heart
NCT03231371	2008	Adenosine	Orthotopic Heart Transplant	Coronary Artery Vasculopathy in Pediatric Heart Transplant Patients
NCT03102125	2019	Regadenoson	Heart Transplant Failure and Rejection	Allograft Dysfunction in Heart Transplant
NCT02597543	2015	Regadenoson	Heart Transplant, Acute Graft Rejection, Chronic Graft Rejection	Stress Cardiac MRI for Evaluation of Nonspecific Allograft Dysfunction
NCT05081115	2021	Adenosine, regadenoson	Coronary Artery Disease, Heart Failure, Hypertrophic Cardiomyopathy	Stress Echo 2030: the Novel ABCDE-(FGLPR) Protocol to Define the Future of Imaging

## Conclusions

In this review, purinergic signaling and its role in IRI and cardiac allograft rejection, as well as clinical applications and prospects were discussed. ATP plays significant roles in inflammation and allograft rejection, whereas adenosine shows anti-inflammatory capabilities and the ability to induce immune tolerance. Balancing ATP and adenosine signaling pathways will be a key factor in regulating immune rejection or immune tolerance, as well as maintaining the long-term survival of heart transplants. Currently, several therapeutic strategies targeting purinergic signaling, such as reducing ATP release or raising levels of adenosine, are available. However, current knowledge on the role of purinergic signaling receptors in heart transplantation remains insufficient. Further studies are required to investigate the maintenance of ATP and adenosine signaling balance, as well as better short- and long-term survival outcomes in patients who underwent heart transplantation.

## Author Contributions

YJ and JL wrote the manuscript. HZ revised the manuscript and figures. PZ designed this project. All authors listed have made a substantial, direct and intellectual contribution to the work, and approved it for publication.

## Funding

This work was supported in part by research grants from the National Key Research and Development Program of China (2018YFA0108700), NSFC Projects of International Cooperation and Exchanges (81720108004), National Natural Science Foundation of China (81974019), the Research Team Project of Natural Science Foundation of Guangdong Province of China (2017A030312007), the key program of guangzhou science research plan (201904020047), the Special Project of Dengfeng Program of Guangdong Provincial People’s Hospital (DFJH201812, KJ012019119, KJ012019423), and the Postdoctoral Sustentation Fund of Guangdong Provincial People’s Hospital (BY012020015).

## Conflict of Interest

The authors declare that this study was conducted in the absence of any commercial or financial relationships that could be construed as a potential conflict of interest.

## Publisher’s Note

All claims expressed in this article are solely those of the authors and do not necessarily represent those of their affiliated organizations, or those of the publisher, the editors and the reviewers. Any product that may be evaluated in this article, or claim that may be made by its manufacturer, is not guaranteed or endorsed by the publisher.

## References

[1] Stehlik J Kobashigawa J Hunt SA Reichenspurner H Kirklin JK. Honoring 50 Years of Clinical Heart Transplantation in Circulation: In-Depth State-Of-the-Art Review. Circulation (2018) 137:71–87. doi: 10.1161/CIRCULATIONAHA.117.029753 29279339

[2] Lund LH Khush KK Cherikh WS Goldfarb S Kucheryavaya AY Levvey BJ. The Registry of the International Society for Heart and Lung Transplantation: Thirty-Fourth Adult Heart Transplantation Report-2017; Focus Theme: Allograft Ischemic Time. J Heart Lung Transplant (2017) 36:1037–46. doi: 10.1016/j.healun.2017.07.019 28779893

[3] Khush KK Hsich E Potena L Cherikh WS Chambers DC Harhay MO. The International Thoracic Organ Transplant Registry of the International Society for Heart and Lung Transplantation: Thirty-Eighth Adult Heart Transplantation Report - 2021; Focus on Recipient Characteristics. J Heart Lung Transplant (2021) 40:1035–49. doi: 10.1016/j.healun.2021.07.015 PMC10282986 34419370

[4] Singh TP Cherikh WS Hsich E Chambers DC Harhay MO Hayes D. The International Thoracic Organ Transplant Registry of the International Society for Heart and Lung Transplantation: Twenty-Fourth Pediatric Heart Transplantation Report — 2021; Focus on Recipient Characteristics. J Heart Lung Transplant (2021) 40:1050–9. doi: 10.1016/j.healun.2021.07.022 PMC10281816 34420853

[5] Zhu Y Lingala B Baiocchi M Arana VT Williams KM Shudo Y. The Stanford Experience of Heart Transplantation Over Five Decades. Eur Heart J (2021) 42(48):4934–43. doi: 10.1093/eurheartj/ehab416 34333595

[6] Rana A Gruessner A Agopian VG Khalpey Z Riaz IB Kaplan B. Survival Benefit of Solid-Organ Transplant in the United States. JAMA Surg (2015) 150:252–9. doi: 10.1001/jamasurg.2014.2038 25629390

[7] Mancini D Goldstein D Taylor S Chen L Gass A DeLair S. Maximizing Donor Allocation: A Review of UNOS Region 9 Donor Heart Turn-Downs. Am J Transplant (2017) 17:3193–8. doi: 10.1111/ajt.14499 28898542

[8] Burnstock G. Purine and Purinergic Receptors. Brain Neurosci Adv (2018) 2:2398212818817494. doi: 10.1177/2398212818817494 32166165 PMC7058212

[9] Idzko M Ferrari D Eltzschig HK. Nucleotide Signalling During Inflammation. Nature (2014) 509:310–7. doi: 10.1038/nature13085 PMC4222675 24828189

[10] Giuliani AL Sarti AC Di Virgilio F. Extracellular Nucleotides and Nucleosides as Signalling Molecules. Immunol Lett (2019) 205:16–24. doi: 10.1016/j.imlet.2018.11.006 30439478

[11] Zeiser R Robson SC Vaikunthanathan T Dworak M Burnstock G. Unlocking the Potential of Purinergic Signaling in Transplantation. Am J Transplant (2016) 16:2781–94. doi: 10.1111/ajt.13801 PMC5472988 27005321

[12] Burnstock G. Purinergic Nerves. Pharmacol Rev (1972) 24:509–81. 4404211

[13] Burnstock G. “A Basis for Distinguishing Two Types of Purinergic Receptor”. In: Straub RW Bolis L, editors. Cell Membrane Receptors for Drugs and Hormones: A Multidisciplinary Approach, vol. 103 . New York: Raven Press (1978). p. 107–18.

[14] Cekic C Linden J. Purinergic Regulation of the Immune System. Nat Rev Immunol (2016) 16:177–92. doi: 10.1038/nri.2016.4 26922909

[15] Yegutkin GG. Enzymes Involved in Metabolism of Extracellular Nucleotides and Nucleosides: Functional Implications and Measurement of Activities. Crit Rev Biochem Mol Biol (2014) 49:473–97. doi: 10.3109/10409238.2014.953627 25418535

[16] Zimmermann H Zebisch M Sträter N. Cellular Function and Molecular Structure of Ecto-Nucleotidases. Purinergic Signal (2012) 8:437–502. doi: 10.1007/s11302-012-9309-4 22555564 PMC3360096

[17] Antonioli L Pacher P Vizi ES Haskó G. CD39 and CD73 in Immunity and Inflammation. Trends Mol Med (2013) 19:355–67. doi: 10.1016/j.molmed.2013.03.005 PMC3674206 23601906

[18] Aymerich I Duflot S Fernández-Veledo S Guillén-Gómez E Huber-Ruano I Casado FJ. The Concentrative Nucleoside Transporter Family (SLC28): New Roles Beyond Salvage? Biochem Soc Trans (2005) 33:216–9. doi: 10.1042/BST0330216 15667311

[19] Young JD. The SLC28 (CNT) and SLC29 (ENT) Nucleoside Transporter Families: A 30-Year Collaborative Odyssey. Biochem Soc Trans (2016) 44:869–76. doi: 10.1042/BST20160038 27284054

[20] Granja T Körner A Glück C Hohmann JD Wang X Köhler D. Targeting CD39 Toward Activated Platelets Reduces Systemic Inflammation and Improves Survival in Sepsis: A Preclinical Pilot Study. Crit Care Med (2019) 47:e420–7. doi: 10.1097/CCM.0000000000003682 30730441

[21] Minor M Alcedo KP Battaglia RA Snider NT. Cell Type- and Tissue-Specific Functions of Ecto-5’-Nucleotidase (CD73). Am J Physiol Cell Physiol (2019) 317:C1079–92. doi: 10.1152/ajpcell.00285.2019 PMC6957383 31461341

[22] Linden J Koch-Nolte F Dahl G. Purine Release, Metabolism, and Signaling in the Inflammatory Response. Annu Rev Immunol (2019) 37:325–47. doi: 10.1146/annurev-immunol-051116-052406 30676821

[23] Roberts V Lu B Rajakumar S Cowan PJ Dwyer KM. The CD39-Adenosinergic Axis in the Pathogenesis of Renal Ischemia–Reperfusion Injury. Purinergic Signal (2013) 9:135–43. doi: 10.1007/s11302-012-9342-3 PMC3646120 23188420

[24] Kishore BK Robson SC Dwyer KM. CD39-Adenosinergic Axis in Renal Pathophysiology and Therapeutics. Purinergic Signal (2018) 14:109–20. doi: 10.1007/s11302-017-9596-x PMC5940625 29332180

[25] Castillo-Leon E Dellepiane S Fiorina P. ATP and T-Cell-Mediated Rejection. Curr Opin Organ Transplant (2018) 23:34–43. doi: 10.1097/MOT.0000000000000484 29120881

[26] Zhong EH Ledderose C De Andrade Mello P Enjyoji K Lunderberg JM Junger W. Structural and Functional Characterization of Engineered Bifunctional Fusion Proteins of CD39 and CD73 Ectonucleotidases. Am J Physiol Cell Physiol (2021) 320:C15–29. doi: 10.1152/ajpcell.00430.2020 PMC7846972 33052071

[27] Giuliani AL Sarti AC Di Virgilio F. Ectonucleotidases in Acute and Chronic Inflammation. Front Pharmacol (2021) 11:619458. doi: 10.3389/fphar.2020.619458 33613285 PMC7887318

[28] Orriss IR Guneri D Hajjawi MOR Shaw K Patel JJ Arnett TR. Activation of the P2Y2 Receptor Regulates Bone Cell Function by Enhancing ATP Release. J Endocrinol (2017) 233:341–56. doi: 10.1530/JOE-17-0042 28420708

[29] Laurain A Rubera I Duranton C Rutsch F Nitschke Y Ray E. Alkaline Phosphatases Account for Low Plasma Levels of Inorganic Pyrophosphate in Chronic Kidney Disease. Front Cell Dev Biol (2020) 8:586831. doi: 10.3389/fcell.2020.586831 33425894 PMC7793922

[30] Quillen EE Haslam GC Samra HS Amani-Taleshi D Knight JA Wyatt DE. Ectoadenylate Kinase and Plasma Membrane ATP Synthase Activities of Human Vascular Endothelial Cells*. J Biol Chem (2006) 281:20728–37. doi: 10.1074/jbc.M513042200 16714292

[31] Burnstock G. Purinergic Receptors in the Heart. Circ Res (1980) 46:175–82. 6247089

[32] Fredholm BB IJzerman AP Jacobson KA Klotz KN Linden J. International Union of Pharmacology. XXV. Nomenclature and Classification of Adenosine Receptors. Pharmacol Rev (2001) 53:527–52. PMC9389454 11734617

[33] Borea PA Gessi S Merighi S Vincenzi F Varani K. Pharmacology of Adenosine Receptors: The State of the Art. Physiol Rev (2018) 98:1591–625. doi: 10.1152/physrev.00049.2017 29848236

[34] Le Duc D Schulz A Lede V Schulze A Thor D Brüser A. P2Y Receptors in Immune Response and Inflammation. Adv Immunol (2017) 136:85–121. doi: 10.1016/bs.ai.2017.05.006 28950952

[35] Jacobson KA Delicado EG Gachet C Kennedy C von Kügelgen I Li B. Update of P2Y Receptor Pharmacology: IUPHAR Review 27. Br J Pharmacol (2020) 177:2413–33. doi: 10.1111/bph.15005 PMC7205808 32037507

[36] Virgilio FD Sarti AC Coutinho-Silva R. Purinergic Signalling, DAMPs and Inflammation. Am J Physiol Cell Physiol (2020) 318:15. doi: 10.1152/ajpcell.00053.2020 32159362

[37] Le T-TT Berg NK Harting MT Li X Eltzschig HK Yuan X. Purinergic Signaling in Pulmonary Inflammation. Front Immunol (2019) 10:1633. doi: 10.3389/fimmu.2019.01633 31379836 PMC6646739

[38] Dos Anjos F Simões JLB Assmann CE Carvalho FB Bagatini MD. Potential Therapeutic Role of Purinergic Receptors in Cardiovascular Disease Mediated by SARS-CoV-2. J Immunol Res (2020) 2020:8632048. doi: 10.1155/2020/8632048 33299899 PMC7709498

[39] Jacobson KA Gao Z-G. Adenosine Receptors as Therapeutic Targets. Nat Rev Drug Discov (2006) 5:247–64. doi: 10.1038/nrd1983 PMC3463109 16518376

[40] Haskó G Antonioli L Cronstein BN. Adenosine Metabolism, Immunity and Joint Health. Biochem Pharmacol (2018) 151:307–13. doi: 10.1016/j.bcp.2018.02.002 PMC5899962 29427624

[41] Tian D Li J Zou L Lin M Shi X Hu Y. Adenosine A1 Receptor Deficiency Aggravates Extracellular Matrix Accumulation in Diabetic Nephropathy Through Disturbance of Peritubular Microenvironment. J Diabetes Res (2021) 2021:5584871. doi: 10.1155/2021/5584871 34671682 PMC8523293

[42] Jain S Barella LF Wess J Reitman ML Jacobson KA. Adenosine A1 Receptor Is Dispensable for Hepatocyte Glucose Metabolism and Insulin Sensitivity. Biochem Pharmacol (2021) 192:114739. doi: 10.1016/j.bcp.2021.114739 34418353 PMC8478863

[43] Shi Y Dai Q Ji B Huang L Zhuang X Mo Y. Electroacupuncture Pretreatment Prevents Cognitive Impairment Induced by Cerebral Ischemia-Reperfusion *via* Adenosine A1 Receptors in Rats. Front Aging Neurosci (2021) 13:680706. doi: 10.3389/fnagi.2021.680706 34413765 PMC8369428

[44] Voors AA Düngen H-D Senni M Nodari S Agostoni P Ponikowski P. Safety and Tolerability of Neladenoson Bialanate, a Novel Oral Partial Adenosine A1 Receptor Agonist, in Patients With Chronic Heart Failure. J Clin Pharmacol (2017) 57:440–51. doi: 10.1002/jcph.828 27624622

[45] Zhang J Han C Dai H Hou J Dong Y Cui X. Hypoxia-Inducible Factor-2α Limits Natural Killer T Cell Cytotoxicity in Renal Ischemia/Reperfusion Injury. J Am Soc Nephrol (2016) 27:92–106. doi: 10.1681/ASN.2014121248 25956511 PMC4696581

[46] Ran H Duan W Gong Z Xu S Zhu H Hou X. Critical Contribution of Adenosine A2A Receptors in Bone Marrow-Derived Cells to White Matter Lesions Induced by Chronic Cerebral Hypoperfusion. J Neuropathol Exp Neurol (2015) 74:305–18. doi: 10.1097/NEN.0000000000000174 25756592

[47] Tang L-M Zhu J-F Wang F Qian J Zhu J Mo Q. Activation of Adenosine A2A Receptor Attenuates Inflammatory Response in a Rat Model of Small-for-Size Liver Transplantation. Transplant Proc (2010) 42:1915–20. doi: 10.1016/j.transproceed.2010.02.084 20620548

[48] Rork TH Wallace KL Kennedy DP Marshall MA Lankford AR Linden J. Adenosine A2A Receptor Activation Reduces Infarct Size in the Isolated, Perfused Mouse Heart by Inhibiting Resident Cardiac Mast Cell Degranulation. Am J Physiol Heart Circ Physiol (2008) 295:H1825–1833. doi: 10.1152/ajpheart.495.2008 PMC2614589 18757481

[49] DiNicolantonio JJ Barroso-Aranda J. Harnessing Adenosine A2A Receptors as a Strategy for Suppressing the Lung Inflammation and Thrombotic Complications of COVID-19: Potential of Pentoxifylline and Dipyridamole. Med Hypotheses (2020) 143:110051. doi: 10.1016/j.mehy.2020.110051 32650197 PMC7330590

[50] Zhang X Du P Luo K Li Y Liu Z Wang W. Hypoxia-Inducible Factor-1alpha Protects the Liver Against Ischemia-Reperfusion Injury by Regulating the A2B Adenosine Receptor. Bioengineered (2021) 12:3737–52. doi: 10.1080/21655979.2021.1953217 PMC8806673 34288817

[51] Dettori I Gaviano L Ugolini F Lana D Bulli I Magni G. Protective Effect of Adenosine A2B Receptor Agonist, BAY60-6583, Against Transient Focal Brain Ischemia in Rat. Front Pharmacol (2020) 11:588757. doi: 10.3389/fphar.2020.588757 33643036 PMC7905306

[52] Mulloy DP Sharma AK Fernandez LG Zhao Y Lau CL Kron IL. Adenosine A3 Receptor Activation Attenuates Lung Ischemia-Reperfusion Injury. Ann Thorac Surg (2013) 95:1762–7. doi: 10.1016/j.athoracsur.2013.01.059 PMC3725313 23541429

[53] Ni Y Liang D Tian Y Kron IL French BA Yang Z. Infarct-Sparing Effect of Adenosine A2B Receptor Agonist Is Primarily Due to Its Action on Splenic Leukocytes *Via* a PI3K/Akt/IL-10 Pathway. J Surg Res (2018) 232:442–9. doi: 10.1016/j.jss.2018.06.042 PMC6251504 30463755

[54] Sui Y Liu J Zhang J Zheng Z Wang Z Jia Z. Expression and Gene Regulation Network of Adenosine Receptor A2B in Lung Adenocarcinoma: A Potential Diagnostic and Prognostic Biomarker. Front Mol Biosci (2021) 8:663011. doi: 10.3389/fmolb.2021.663011 34350210 PMC8326519

[55] Ohana G Bar-Yehuda S Arich A Madi L Dreznick Z Rath-Wolfson L. Inhibition of Primary Colon Carcinoma Growth and Liver Metastasis by the A3 Adenosine Receptor Agonist CF101. Br J Cancer (2003) 89:1552–8. doi: 10.1038/sj.bjc.6601315 PMC2394357 14562031

[56] Bar-Yehuda S Madi L Silberman D Gery S Shkapenuk M Fishman P. CF101, an Agonist to the A3 Adenosine Receptor, Enhances the Chemotherapeutic Effect of 5-Fluorouracil in a Colon Carcinoma Murine Model. Neoplasia (2005) 7:85–90. doi: 10.1593/neo.04364 15720820 PMC1490317

[57] Burnstock G. P2X Ion Channel Receptors and Inflammation. Purinergic Signal (2016) 12:59–67. doi: 10.1007/s11302-015-9493-0 26739702 PMC4749528

[58] Yeudall S Leitinger N Laubach VE. Extracellular Nucleotide Signaling in Solid Organ Transplantation. Am J Transplant (2020) 20:633–40. doi: 10.1111/ajt.15651 PMC7042041 31605463

[59] Vassort G. Adenosine 5’-Triphosphate: A P2-Purinergic Agonist in the Myocardium. Physiol Rev (2001) 81:767–806. doi: 10.1152/physrev.2001.81.2.767 11274344

[60] Kristiansen SB Skovsted GF Berchtold LA Radziwon-Balicka A Dreisig K Edvinsson L. Role of Pannexin and Adenosine Triphosphate (ATP) Following Myocardial Ischemia/Reperfusion. Scand Cardiovasc J (2018) 52:340–3. doi: 10.1080/14017431.2018.1552793 30481075

[61] Lewis CJ Evans RJ. P2X Receptor Immunoreactivity in Different Arteries From the Femoral, Pulmonary, Cerebral, Coronary and Renal Circulations. J Vasc Res (2001) 38:332–40. doi: 10.1159/000051064 11455204

[62] Mahaut-Smith MP Jones S Evans RJ. The P2X1 Receptor and Platelet Function. Purinergic Signalling (2011) 7:341–56. doi: 10.1007/s11302-011-9224-0 PMC3166991 21484087

[63] Kudira R Malinka T Kohler A Dosch M de Agüero MG Melin N. P2X1-Regulated IL-22 Secretion by Innate Lymphoid Cells Is Required for Efficient Liver Regeneration. Hepatology (2016) 63:2004–17. doi: 10.1002/hep.28492 26853442

[64] Zhang C Li G Liang S Xu C Zhu G Wang Y. Myocardial Ischemic Nociceptive Signaling Mediated by P2X3 Receptor in Rat Stellate Ganglion Neurons. Brain Res Bull (2008) 75:77–82. doi: 10.1016/j.brainresbull.2007.07.031 18158099

[65] Xu X Liu B Yang J Zou Y Sun M Li Z. Glucokinase in Stellate Ganglia Cooperates With P2X3 Receptor to Develop Cardiac Sympathetic Neuropathy in Type 2 Diabetes Rats. Brain Res Bull (2020) 165:290–7. doi: 10.1016/j.brainresbull.2020.10.004 33091480

[66] Srivastava P Cronin CG Scranton VL Jacobson KA Liang BT Verma R. Neuroprotective and Neuro-Rehabilitative Effects of Acute Purinergic Receptor P2X4 (P2X4R) Blockade After Ischemic Stroke. Exp Neurol (2020) 329:113308. doi: 10.1016/j.expneurol.2020.113308 32289314 PMC7242087

[67] Verma R Cronin CG Hudobenko J Venna VR McCullough LD Liang BT. Deletion of the P2X4 Receptor Is Neuroprotective Acutely, But Induces a Depressive Phenotype During Recovery From Ischemic Stroke. Brain Behav Immun (2017) 66:302–12. doi: 10.1016/j.bbi.2017.07.155 PMC5650951 28751018

[68] D’Addio F Vergani A Potena L Maestroni A Usuelli V Ben Nasr M. P2X7R Mutation Disrupts the NLRP3-Mediated Th Program and Predicts Poor Cardiac Allograft Outcomes. J Clin Invest (2018) 128:3490–503. doi: 10.1172/JCI94524 PMC6063506 30010623

[69] Di Virgilio F Sarti AC Falzoni S De Marchi E Adinolfi E. Extracellular ATP and P2 Purinergic Signalling in the Tumour Microenvironment. Nat Rev Cancer (2018) 18:601–18. doi: 10.1038/s41568-018-0037-0 30006588

[70] Woods LT Forti KM Shanbhag VC Camden JM Weisman GA. P2Y Receptors for Extracellular Nucleotides: Contributions to Cancer Progression and Therapeutic Implications. Biochem Pharmacol (2021) 187:114406. doi: 10.1016/j.bcp.2021.114406 33412103 PMC8096679

[71] Zuo X Li Q Ya F Ma L-J Tian Z Zhao M. Ginsenosides Rb2 and Rd2 Isolated From Panax Notoginseng Flowers Attenuate Platelet Function Through P2Y12-Mediated cAMP/PKA and PI3K/Akt/Erk1/2 Signaling. Food Funct (2021) 12:5793–805. doi: 10.1039/d1fo00531f 34041517

[72] Sophocleous RA Miles NA Ooi L Sluyter R. P2Y2 and P2X4 Receptors Mediate Ca2+ Mobilization in DH82 Canine Macrophage Cells. Int J Mol Sci (2020) 21:E8572. doi: 10.3390/ijms21228572 33202978 PMC7696671

[73] Mesto N Bailbe D Eskandar M Pommier G Gil S Tolu S. Involvement of P2Y Signaling in the Restoration of Glucose-Induced Insulin Exocytosis in Pancreatic β Cells Exposed to Glucotoxicity. J Cell Physiol (2021) 237(1):881–96. doi: 10.1002/jcp.30564 34435368

[74] Magni G Ceruti S. The Role of Adenosine and P2Y Receptors Expressed by Multiple Cell Types in Pain Transmission. Brain Res Bull (2019) 151:132–43. doi: 10.1016/j.brainresbull.2019.02.011 30797817

[75] Perera LMB Sekiguchi A Uchiyama A Uehara A Fujiwara C Yamazaki S. The Regulation of Skin Fibrosis in Systemic Sclerosis by Extracellular ATP *via* P2Y2 Purinergic Receptor. J Invest Dermatol (2019) 139:890–9. doi: 10.1016/j.jid.2018.10.027 30404019

[76] Djerada Z Feliu C Richard V Millart H. Current Knowledge on the Role of P2Y Receptors in Cardioprotection Against Ischemia-Reperfusion. Pharmacol Res (2017) 118:5–18. doi: 10.1016/j.phrs.2016.08.009 27520402

[77] Koizumi S Shigemoto-Mogami Y Nasu-Tada K Shinozaki Y Ohsawa K Tsuda M. UDP Acting at P2Y6 Receptors Is a Mediator of Microglial Phagocytosis. Nature (2007) 446:1091–5. doi: 10.1038/nature05704 PMC3464483 17410128

[78] Elliott MR Koster KM Murphy PS. Efferocytosis Signaling in the Regulation of Macrophage Inflammatory Responses. J Immunol (2017) 198:1387–94. doi: 10.4049/jimmunol.1601520 PMC5301545 28167649

[79] Chen Y Corriden R Inoue Y Yip L Hashiguchi N Zinkernagel A. ATP Release Guides Neutrophil Chemotaxis Via P2Y2 A3 Receptors. Science (2006) 314:1792–5. doi: 10.1126/science.1132559 17170310

[80] Liverani E Rico MC Yaratha L Tsygankov AY Kilpatrick LE Kunapuli SP. LPS-Induced Systemic Inflammation is More Severe in P2Y12 Null Mice. J Leukoc Biol (2014) 95:313–23. doi: 10.1189/jlb.1012518 PMC4051260 24142066

[81] Liverani E Rico MC Tsygankov AY Kilpatrick LE Kunapuli SP. P2Y12 Receptor Modulates Sepsis-Induced Inflammation. Arterioscler Thromb Vasc Biol (2016) 36:961–71. doi: 10.1161/ATVBAHA.116.307401 PMC4850113 27055904

[82] Lovászi M Branco Haas C Antonioli L Pacher P Haskó G. The Role of P2Y Receptors in Regulating Immunity and Metabolism. Biochem Pharmacol (2021) 187:114419. doi: 10.1016/j.bcp.2021.114419 33460626

[83] Cohen R Shainberg A Hochhauser E Cheporko Y Tobar A Birk E. UTP Reduces Infarct Size and Improves Mice Heart Function After Myocardial Infarct *via* P2Y2 Receptor. Biochem Pharmacol (2011) 82:1126–33. doi: 10.1016/j.bcp.2011.07.094 21839729

[84] Liverani E Kilpatrick LE Tsygankov AY Kunapuli SP. The Role of P2Y_12_ Receptor and Activated Platelets During Inflammation. Curr Drug Targets (2014) 15:720–8. doi: 10.2174/1389450115666140519162133 PMC5681251 24845219

[85] Certal M Vinhas A Pinheiro AR Ferreirinha F Barros-Barbosa AR Silva I. Calcium Signaling and the Novel Anti-Proliferative Effect of the UTP-Sensitive P2Y11 Receptor in Rat Cardiac Myofibroblasts. Cell Calcium (2015) 58:518–33. doi: 10.1016/j.ceca.2015.08.004 26324417

[86] Sunggip C Nishimura A Shimoda K Numaga-Tomita T Tsuda M Nishida M. Purinergic P2Y6 Receptors: A New Therapeutic Target of Age-Dependent Hypertension. Pharmacol Res (2017) 120:51–9. doi: 10.1016/j.phrs.2017.03.013 28336370

[87] Clouet S Di Pietrantonio L Daskalopoulos E-P Esfahani H Horckmans M Vanorlé M. Loss of Mouse P2Y6 Nucleotide Receptor Is Associated With Physiological Macrocardia and Amplified Pathological Cardiac Hypertrophy. J Biol Chem (2016) 291:15841–52. doi: 10.1074/jbc.M115.684118 PMC4957065 27231349

[88] Lemaire A Vanorlé M Horckmans M di Pietrantonio L Clouet S Robaye B. Mouse P2Y4 Nucleotide Receptor Is a Negative Regulator of Cardiac Adipose-Derived Stem Cell Differentiation and Cardiac Fat Formation. Stem Cells Dev (2017) 26:363–73. doi: 10.1089/scd.2016.0166 27855539

[89] Springthorpe B Bailey A Barton P Birkinshaw TN Bonnert RV Brown RC. From ATP to AZD6140: The Discovery of an Orally Active Reversible P2Y12 Receptor Antagonist for the Prevention of Thrombosis. Bioorg Med Chem Lett (2007) 17:6013–8. doi: 10.1016/j.bmcl.2007.07.057 17827008

[90] Coukell AJ Markham A. Clopidogrel. Drugs (1997) 54:745–750; discussion 751. doi: 10.2165/00003495-199754050-00006 9360060

[91] Chambers DC Yusen RD Cherikh WS Goldfarb SB Kucheryavaya AY Khusch K. The Registry of the International Society for Heart and Lung Transplantation: Thirty-Fourth Adult Lung And Heart-Lung Transplantation Report-2017; Focus Theme: Allograft Ischemic Time. J Heart Lung Transplant (2017) 36:1047–59. doi: 10.1016/j.healun.2017.07.016 28784324

[92] Rivard AL Gallegos R Ogden IM Bianco RW. Perfusion Preservation of the Donor Heart: Basic Science to Pre-Clinical. J Extra Corpor Technol (2009) 41:140–8. PMC4679948 19806796

[93] Frye CC Bery AI Kreisel D Kulkarni HS. Sterile Inflammation in Thoracic Transplantation. Cell Mol Life Sci (2021) 78:581–601. doi: 10.1007/s00018-020-03615-7 32803398 PMC7878195

[94] Di Virgilio F Sarti AC Coutinho-Silva R. Purinergic Signaling, DAMPs, and Inflammation. Am J Physiol Cell Physiol (2020) 318:C832–5. doi: 10.1152/ajpcell.00053.2020 32159362

[95] Kopp R Krautloher A Ramírez-Fernández A Nicke A. P2X7 Interactions and Signaling – Making Head or Tail of It. Front Mol Neurosci (2019) 12:183. doi: 10.3389/fnmol.2019.00183 31440138 PMC6693442

[96] Shokoples BG Paradis P Schiffrin EL. P2X7 Receptors. Arterioscler Thromb Vasc Biol (2021) 41:186–99. doi: 10.1161/ATVBAHA.120.315116 PMC7752223 32998520

[97] Kawaguchi M Takahashi M Hata T Kashima Y Usui F Morimoto H. Inflammasome Activation of Cardiac Fibroblasts Is Essential for Myocardial Ischemia/Reperfusion Injury. Circulation (2011) 123:594–604. doi: 10.1161/CIRCULATIONAHA.110.982777 21282498

[98] Pomerantz BJ Reznikov LL Harken AH Dinarello CA. Inhibition of Caspase 1 Reduces Human Myocardial Ischemic Dysfunction *via* Inhibition of IL-18 and IL-1beta. Proc Natl Acad Sci USA (2001) 98:2871–6. doi: 10.1073/pnas.041611398 PMC30232 11226333

[99] Hesse J Leberling S Boden E Friebe D Schmidt T Ding Z. CD73-Derived Adenosine and Tenascin-C Control Cytokine Production by Epicardium-Derived Cells Formed After Myocardial Infarction. FASEB J (2017) 31:3040–53. doi: 10.1096/fj.201601307R 28363952

[100] Sandanger Ø Ranheim T Vinge LE Bliksøen M Alfsnes K Finsen AV. The NLRP3 Inflammasome is Up-Regulated in Cardiac Fibroblasts and Mediates Myocardial Ischaemia-Reperfusion Injury. Cardiovasc Res (2013) 99:164–74. doi: 10.1093/cvr/cvt091 23580606

[101] Vessey DA Li L Kelley M. Pannexin-I/P2X 7 Purinergic Receptor Channels Mediate the Release of Cardioprotectants Induced by Ischemic Pre- and Postconditioning. J Cardiovasc Pharmacol Ther (2010) 15:190–5. doi: 10.1177/1074248409360356 20200324

[102] Vessey DA Li L Kelley M. P2X7 Receptor Agonists Pre- and Postcondition the Heart Against Ischemia-Reperfusion Injury by Opening Pannexin-1/P2X_7_ Channels. Am J Physiol Heart Circ Physiol (2011) 301:H881–887. doi: 10.1152/ajpheart.00305.2011 21685263

[103] Vessey DA Li L Kelley M. Ischemic Preconditioning Requires Opening of Pannexin-1/P2X(7) Channels Not Only During Preconditioning But Again After Index Ischemia at Full Reperfusion. Mol Cell Biochem (2011) 351:77–84. doi: 10.1007/s11010-011-0713-9 21267638

[104] Dost T. Cardioprotective Properties of the Platelet P2Y12 Receptor Inhibitor Prasugrel on Cardiac Ischemia/Reperfusion Injury. Pharmacol Rep (2020) 72:672–9. doi: 10.1007/s43440-019-00046-5 32048257

[105] Hjortbak MV Olesen KKW Seefeldt JM Lassen TR Jensen RV Perkins A. Translation of Experimental Cardioprotective Capability of P2Y12 Inhibitors Into Clinical Outcome in Patients With ST-Elevation Myocardial Infarction. Basic Res Cardiol (2021) 116:36. doi: 10.1007/s00395-021-00870-y 34037861

[106] Onorati F Dobson GP Biagio LS Abbasciano R Fanti D Covajes C. Superior Myocardial Protection Using “Polarizing” Adenosine, Lidocaine, and Mg2+ Cardioplegia in Humans. J Am Coll Cardiol (2016) 67:1751–3. doi: 10.1016/j.jacc.2015.12.071 27056783

[107] Francica A Tonelli F Rossetti C Tropea I Luciani GB Faggian G. Cardioplegia Between Evolution and Revolution: From Depolarized to Polarized Cardiac Arrest in Adult Cardiac Surgery. JCM (2021) 10:4485. doi: 10.3390/jcm10194485 34640503 PMC8509840

[108] Cw W A A D H B X Y L P M. A Cardioprotective Preservation Strategy Employing *Ex Vivo* Heart Perfusion Facilitates Successful Transplant of Donor Hearts After Cardiocirculatory Death. J Heart Lung Transplant (2013) 32(7):734–43. doi: 10.1016/j.healun.2013.04.016 23796155

[109] Méndez-Carmona N Wyss RK Arnold M Segiser A Kalbermatter N Joachimbauer A. Effects of Graft Preservation Conditions on Coronary Endothelium and Cardiac Functional Recovery in a Rat Model of Donation After Circulatory Death. J Heart Lung Transplant (2021) 40(11):1396–407. doi: 10.1016/j.healun.2021.07.028 34509349

[110] White CW Ambrose E Müller A Li Y Le H Thliveris J. Avoidance of Profound Hypothermia During Initial Reperfusion Improves the Functional Recovery of Hearts Donated After Circulatory Death. Am J Transplant (2016) 16:773–82. doi: 10.1111/ajt.13574 26780159

[111] Sanjani MS Teng B Krahn T Tilley S Ledent C Mustafa SJ. Contributions of A2A and A2B Adenosine Receptors in Coronary Flow Responses in Relation to the KATP Channel Using A2B and A2A/2B Double-Knockout Mice. Am J Physiol Heart Circ Physiol (2011) 301:H2322–2333. doi: 10.1152/ajpheart.00052.2011 PMC3233816 21949117

[112] Shryock JC Snowdy S Baraldi PG Cacciari B Spalluto G Monopoli A. A2A-Adenosine Receptor Reserve for Coronary Vasodilation. Circulation (1998) 98:711–8. doi: 10.1161/01.cir.98.7.711 9715864

[113] Berwick ZC Payne GA Lynch B Dick GM Sturek M Tune JD. Contribution of Adenosine A(2A) and A(2B) Receptors to Ischemic Coronary Dilation: Role of K(V) and K(ATP) Channels. Microcirculation (2010) 17:600–7. doi: 10.1111/j.1549-8719.2010.00054.x PMC3051166 21044214

[114] Eltzschig HK Bonney SK Eckle T. Attenuating Myocardial Ischemia by Targeting A2B Adenosine Receptors. Trends Mol Med (2013) 19:345–54. doi: 10.1016/j.molmed.2013.02.005 PMC3674126 23540714

[115] Koeppen M Eckle T Eltzschig HK. Interplay of Hypoxia and A2B Adenosine Receptors in Tissue Protection. Adv Pharmacol (2011) 61:145–86. doi: 10.1016/B978-0-12-385526-8.00006-0 21586359

[116] Laxson DD Homans DC Bache RJ. Inhibition of Adenosine-Mediated Coronary Vasodilation Exacerbates Myocardial Ischemia During Exercise. Am J Physiol (1993) 265:H1471–1477. doi: 10.1152/ajpheart.1993.265.5.H1471 8238557

[117] Eckle T Hartmann K Bonney S Reithel S Mittelbronn M Walker LA. Adora2b-Elicited Per2 Stabilization Promotes a HIF-Dependent Metabolic Switch Crucial for Myocardial Adaptation to Ischemia. Nat Med (2012) 18:774–82. doi: 10.1038/nm.2728 PMC3378044 22504483

[118] Reichelt ME Shanu A Willems L Witting PK Ellis NA Blackburn MR. Endogenous Adenosine Selectively Modulates Oxidant Stress *via* the A1 Receptor in Ischemic Hearts. Antioxid Redox Signal (2009) 11:2641–50. doi: 10.1089/ars.2009.2644 PMC2861535 19552606

[119] Rothermel BA Hill JA. Adenosine A3 Receptor and Cardioprotection: Enticing, Enigmatic, Elusive. Circulation (2008) 118:1691–3. doi: 10.1161/CIRCULATIONAHA.108.810101 PMC2612935 18936336

[120] Wolff G Truse R Decking U. Extracellular Adenosine Formation by Ecto-5’-Nucleotidase (CD73) Is No Essential Trigger for Early Phase Ischemic Preconditioning. PLoS One (2015) 10:e0135086. doi: 10.1371/journal.pone.0135086 26261991 PMC4532361

[121] Smith SB Xu Z Novitskaya T Zhang B Chepurko E Pu X-A. Impact of Cardiac-Specific Expression of CD39 on Myocardial Infarct Size in Mice. Life Sci (2017) 179:54–9. doi: 10.1016/j.lfs.2016.10.016 27756600

[122] Wheeler DG Joseph ME Mahamud SD Aurand WL Mohler PJ Pompili VJ. Transgenic Swine: Expression of Human CD39 Protects Against Myocardial Injury. J Mol Cell Cardiol (2012) 52:958–61. doi: 10.1016/j.yjmcc.2012.01.002 PMC3327755 22269791

[123] Bönner F Borg N Burghoff S Schrader J. Resident Cardiac Immune Cells and Expression of the Ectonucleotidase Enzymes CD39 and CD73 After Ischemic Injury. PLoS One (2012) 7:e34730. doi: 10.1371/journal.pone.0034730 22514659 PMC3326036

[124] Köhler D Eckle T Faigle M Grenz A Mittelbronn M Laucher S. CD39/Ectonucleoside Triphosphate Diphosphohydrolase 1 Provides Myocardial Protection During Cardiac Ischemia/Reperfusion Injury. Circulation (2007) 116:1784–94. doi: 10.1161/CIRCULATIONAHA.107.690180 17909107

[125] Ziegler M Hohmann JD Searle AK Abraham M-K Nandurkar HH Wang X. Peter K. A Single-Chain Antibody-CD39 Fusion Protein Targeting Activated Platelets Protects From Cardiac Ischaemia/Reperfusion Injury. Eur Heart J (2018) 39:111–6. doi: 10.1093/eurheartj/ehx218 28472483

[126] Dzeja PP Bast P Pucar D Wieringa B Terzic A. Defective Metabolic Signaling in Adenylate Kinase AK1 Gene Knock-Out Hearts Compromises Post-Ischemic Coronary Reflow. J Biol Chem (2007) 282:31366–72. doi: 10.1074/jbc.M705268200 PMC3232003 17704060

[127] Pucar D Bast P Gumina RJ Lim L Drahl C Juranic N. Adenylate Kinase AK1 Knockout Heart: Energetics and Functional Performance Under Ischemia-Reperfusion. Am J Physiol Heart Circ Physiol (2002) 283:H776–782. doi: 10.1152/ajpheart.00116.2002 12124227

[128] Vergani A Tezza S D’Addio F Fotino C Liu K Niewczas M. Long-Term Heart Transplant Survival by Targeting the Ionotropic Purinergic Receptor P2X7. Circulation (2013) 127:463–75. doi: 10.1161/CIRCULATIONAHA.112.123653 PMC3569513 23250993

[129] Wu C Zhao Y Xiao X Fan Y Kloc M Liu W. Graft-Infiltrating Macrophages Adopt an M2 Phenotype and Are Inhibited by Purinergic Receptor P2X7 Antagonist in Chronic Rejection. Am J Transplant (2016) 16:2563–73. doi: 10.1111/ajt.13808 PMC5552361 27575724

[130] Benoist L Chadet S Genet T Lefort C Heraud A Danila MD. Stimulation of P2Y11 Receptor Protects Human Cardiomyocytes Against Hypoxia/Reoxygenation Injury and Involves Pkcϵ Signaling Pathway. Sci Rep (2019) 9:11613. doi: 10.1038/s41598-019-48006-6 31406184 PMC6690895

[131] Bourguignon T Benoist L Chadet S Miquelestorena-Standley E Fromont G Ivanes F. Stimulation of Murine P2Y11-Like Purinoreceptor Protects Against Hypoxia/Reoxygenation Injury and Decreases Heart Graft Rejection Lesions. J Thorac Cardiovasc Surg (2019) 158:780–90.e1. doi: 10.1016/j.jtcvs.2018.12.014 30711276

[132] Imai M Takigami K Guckelberger O Kaczmarek E Csizmadia E Bach FH. Recombinant Adenoviral Mediated CD39 Gene Transfer Prolongs Cardiac Xenograft Survival. Transplantation (2000) 70:864–70. doi: 10.1097/00007890-200009270-00003 11014639

[133] Koyamada N Miyatake T Candinas D Hechenleitner P Siegel J Hancock WW. Apyrase Administration Prolongs Discordant Xenograft Survival. Transplantation (1996) 62:1739–43. doi: 10.1097/00007890-199612270-00008 8990354

[134] Dwyer KM Robson SC Nandurkar HH Campbell DJ Gock H Murray-Segal LJ. Thromboregulatory Manifestations in Human CD39 Transgenic Mice and the Implications for Thrombotic Disease and Transplantation. J Clin Invest (2004) 113:1440–6. doi: 10.1172/JCI200419560 PMC406523 15146241

[135] Hasegawa T Bouïs D Liao H Visovatti SH Pinsky DJ. Ecto-5’ Nucleotidase (CD73)-Mediated Adenosine Generation and Signaling in Murine Cardiac Allograft Vasculopathy. Circ Res (2008) 103:1410–21. doi: 10.1161/CIRCRESAHA.108.180059 PMC3644954 19008478

[136] Hu Y Kong D Qin Y Yu D Jin W Li X. CD73 Expression is Critical to Therapeutic Effects of Human Endometrial Regenerative Cells in Inhibition of Cardiac Allograft Rejection in Mice. Stem Cells Trans Med (2021) 10:465–78. doi: 10.1002/sctm.20-0154 PMC7900594 33124777

[137] Ngamjariyawat A Turpaev K Vasylovska S Kozlova EN Welsh N. Co-Culture of Neural Crest Stem Cells (NCSC) and Insulin Producing Beta-TC6 Cells Results in Cadherin Junctions and Protection Against Cytokine-Induced Beta-Cell Death. PLoS One (2013) 8:e61828. doi: 10.1371/journal.pone.0061828 23613946 PMC3629122

[138] Dong S Zhang K Shi Y. Carbenoxolone has the Potential to Ameliorate Acute Incision Pain in Rats. Mol Med Rep (2021) 24:520. doi: 10.3892/mmr.2021.12159 34013377 PMC8160483

[139] Ma D Feng L Cheng Y Xin M You J Yin X. Astrocytic Gap Junction Inhibition by Carbenoxolone Enhances the Protective Effects of Ischemic Preconditioning Following Cerebral Ischemia. J Neuroinflamm (2018) 15:198. doi: 10.1186/s12974-018-1230-5 PMC6034345 29976213

[140] Sharma AK Charles EJ Zhao Y Narahari AK Baderdinni PK Good ME. Pannexin-1 Channels on Endothelial Cells Mediate Vascular Inflammation During Lung Ischemia-Reperfusion Injury. Am J Physiol Lung Cell Mol Physiol (2018) 315:L301–12. doi: 10.1152/ajplung.00004.2018 PMC6139659 29745255

[141] Ferenbach DA Sheldrake TA Dhaliwal K Kipari TMJ Marson LP Kluth DC. Macrophage/monocyte Depletion by Clodronate, But Not Diphtheria Toxin, Improves Renal Ischemia/Reperfusion Injury in Mice. Kidney Int (2012) 82:928–33. doi: 10.1038/ki.2012.207 22673886

[142] Li Z Xu X Feng X Murphy PM. The Macrophage-Depleting Agent Clodronate Promotes Durable Hematopoietic Chimerism and Donor-Specific Skin Allograft Tolerance in Mice. Sci Rep (2016) 6:22143. doi: 10.1038/srep22143 26917238 PMC4768260

[143] Wu YL Ye Q Eytan DF Liu L Rosario BL Hitchens TK. Magnetic Resonance Imaging Investigation of Macrophages in Acute Cardiac Allograft Rejection After Heart Transplantation. Circ Cardiovasc Imaging (2013) 6:965–73. doi: 10.1161/CIRCIMAGING.113.000674 PMC3886711 24097421

[144] Abraham M-K Jost E Hohmann JD Searle AK Bongcaron V Song Y. A Recombinant Fusion Construct Between Human Serum Albumin and NTPDase CD39 Allows Anti-Inflammatory and Anti-Thrombotic Coating of Medical Devices. Pharmaceutics (2021) 13:1504. doi: 10.3390/pharmaceutics13091504 34575580 PMC8466136

[145] Savi P Labouret C Delesque N Guette F Lupker J Herbert JM. P2y(12), a New Platelet ADP Receptor, Target of Clopidogrel. Biochem Biophys Res Commun (2001) 283:379–83. doi: 10.1006/bbrc.2001.4816 11327712

[146] Kaza EA Egalka MC Zhou H Chen J Evans D Prats J. P2Y12 Receptor Function and Response to Cangrelor in Neonates With Cyanotic Congenital Heart Disease. JACC Basic Transl Sci (2017) 2:465–76. doi: 10.1016/j.jacbts.2017.04.002 PMC5646421 29057376

[147] Saemann L Korkmaz-Icöz S Hoorn F Veres G Kraft P Georgevici A-I. Reconditioning of Circulatory Death Hearts by *Ex-Vivo* Machine Perfusion With a Novel HTK-N Preservation Solution. J Heart Lung Transplant (2021) 40:1135–44. doi: 10.1016/j.healun.2021.07.009 34420849

[148] Yang C Xu H Cai L Du X Jiang Y Zhang Y. Donor Pretreatment With Adenosine Monophosphate-Activated Protein Kinase Activator Protects Cardiac Grafts From Cold Ischaemia/Reperfusion Injury. Eur J Cardiothorac Surg (2016) 49:1354–60. doi: 10.1093/ejcts/ezv413 26609046

[149] Korkmaz-Icöz S Radovits T Loganathan S Li S Ruppert M Benke K. Prolonging Hypothermic Ischaemic Cardiac and Vascular Storage by Inhibiting the Activation of the Nuclear Enzyme Poly(Adenosine Diphosphate-Ribose) Polymerase. Eur J Cardiothorac Surg (2017) 51:829–35. doi: 10.1093/ejcts/ezw426 28204209

[150] Joglar JA Wan EY Chung MK Gutierrez A Slaughter MS Bateson BP. Management of Arrhythmias After Heart Transplant: Current State and Considerations for Future Research. Circ Arrhythm Electrophysiol (2021) 14:e007954. doi: 10.1161/CIRCEP.120.007954 33685207

[151] Flyer JN Zuckerman WA Richmond ME Anderson BR Mendelsberg TG McAllister JM. Prospective Study of Adenosine on Atrioventricular Nodal Conduction in Pediatric and Young Adult Patients After Heart Transplantation. Circulation (2017) 135:2485–93. doi: 10.1161/CIRCULATIONAHA.117.028087 PMC5539538 28450351

[152] Madani MH Canter CE Balzer DT Watkins MP Wickline SA. Noninvasive Detection of Transplant Coronary Artery Disease With Contrast-Enhanced Cardiac MRI in Pediatric Cardiac Transplants. J Heart Lung Transplant (2012) 31:1234–5. doi: 10.1016/j.healun.2012.06.001 PMC3665003 22749830

[153] Duran SR Huffaker T Dixon B Gooty V Abou Zahr R Arar Y. Feasibility and Safety of Quantitative Adenosine Stress Perfusion Cardiac Magnetic Resonance Imaging in Pediatric Heart Transplant Patients With and Without Coronary Allograft Vasculopathy. Pediatr Radiol (2021) 51:1311–21. doi: 10.1007/s00247-021-04977-1 33791838

[154] Voors AA Bax JJ Hernandez AF Wirtz AB Pap AF Ferreira AC. PANTHEON Investigators. Safety and Efficacy of the Partial Adenosine A1 Receptor Agonist Neladenoson Bialanate in Patients With Chronic Heart Failure With Reduced Ejection Fraction: A Phase IIb, Randomized, Double-Blind, Placebo-Controlled Trial. Eur J Heart Fail (2019) 21:1426–33. doi: 10.1002/ejhf.1591 31523892

[155] Shah SJ Voors AA McMurray JJV Kitzman DW Viethen T Bomfim Wirtz A. Effect of Neladenoson Bialanate on Exercise Capacity Among Patients With Heart Failure With Preserved Ejection Fraction: A Randomized Clinical Trial. JAMA (2019) 321:2101–12. doi: 10.1001/jama.2019.6717 PMC6549300 31162568

[156] Emaminia A Lapar DJ Zhao Y Steidle JF Harris DA Laubach VE. Adenosine a_2_A Agonist Improves Lung Function During *Ex-Vivo* Lung Perfusion. Ann Thorac Surg (2011) 92:1840–6. doi: 10.1016/j.athoracsur.2011.06.062 PMC3259746 22051279

[157] Lau CL Beller JP Boys JA Zhao Y Phillips J Cosner M. Adenosine A2A Receptor Agonist (Regadenoson) in Human Lung Transplantation. J Heart Lung Transplant (2020) 39:563–70. doi: 10.1016/j.healun.2020.02.003 32503727

